# Pneumonectomy following penetrating trauma with ECMO as postoperative support: case report – (Lung trauma and ECMO)

**DOI:** 10.1186/s13019-024-02862-0

**Published:** 2024-07-15

**Authors:** Álvaro Ignacio Sánchez-Ortiz, Diego Peña-González, Alberto F. García, Diego Fernando Bautista-Rincón, Carlos Alejandro García-González, Alejandro Moreno-Angarita, Astrid Carolina Álvarez-Ortega, Nicolas Felipe Torres-España, Eduardo Alberto Cadavid-Alvear, Mauricio Velásquez-Galvis

**Affiliations:** 1 Department of Thoracic Surgery, Fundación Valle del Lili, Kra 98 No. 18-49, Cali, 760032 Colombia; 2 Department of Cardiovascular Surgery, Fundación Valle del Lili, Kra 98 No. 18-49, Cali, Colombia; 3 Intensive Care Unit, Fundación Valle del Lili, Kra 98 No. 18-49, Cali, Colombia; 4 Department of Trauma Surgery, Fundación Valle del Lili, Kra 98 No. 18-49, Cali, Colombia; 5 Department of Radiology and Diagnostic Imaging, Fundación Valle del Lili, Kra 98 No. 18-49, Cali, Colombia; 6 Clinical Research Center, Fundación Valle del Lili, Kra 98 No. 18-49, Cali, Colombia

**Keywords:** ECMO, Traumatic pneumonectomy, Case report, Penetrating thoracic injury

## Abstract

**Background:**

Penetrating thoracic injuries have a significant risk of morbi-mortality. Despite the advancements in damage control methods, a subset of patients with severe pulmonary vascular lesions and bronchial injuries persists. In some of these cases, post-traumatic pneumonectomy is required, and perioperative extracorporeal membrane oxygenation (ECMO) support may be required due to right ventricular failure and respiratory failure.

**Case description:**

A male was brought to the emergency department (ED) with a penetrating thoracic injury, presenting with massive right hemothorax and active bleeding that required ligation of the right pulmonary hilum to control the bleeding. Subsequently, he developed right ventricular dysfunction and ARDS, necessitating a dynamic hybrid ECMO configuration to support his condition and facilitate recovery.

**Conclusions:**

Penetrating thoracic injuries with severe pulmonary vascular lesions may need pneumonectomy to control bleeding. ECMO support reduces the associated mortality by decreasing the complications rate. A multidisciplinary team is essential to achieve good outcomes in severe compromised patients.

## Background

The reported incidence of lung resection in traumatic injuries is 0.08% [1]. Pneumonectomy is performed even less commonly, occurring at an incidence of 0.01% amongst trauma patients. Traumatic pneumonectomy (TP) is a rare but critical intervention in thoracic trauma, with a mortality rate ranging from 50 to 100% [1]. A complete pneumonectomy may be required to control hilar hemorrhage or if pulmonary and bronchial injuries are beyond repair. However, the mortality rates of TP are high. Wagner et al. suggested rapid pneumonectomy for unstable patients with central hilar vascular destruction [2]. Urgent TP is typically conducted following hemorrhage from major hilar or bronchial injuries where lung salvage is unfeasible [1, 3, 4]. Early post-TP fatalities can result from acute right heart failure due to a sudden increase in pulmonary artery pressure. This strain on the thin-walled right ventricle leads to subsequent left ventricular dysfunction [1]. Right heart failure and pulmonary edema in the remaining lung tissue can manifest within hours post-pneumonectomy [5]. However, in extremis patients a more aggressive and timely initiation of this procedure should be warranted [1].

The use of extracorporeal membrane oxygenation (ECMO) in trauma patients can serve as an adjunct therapy when conventional treatments are ineffective. The indications for ECMO in trauma cases, however, remain uncertain, and clinical outcomes vary due to the need for anticoagulation, hemorrhage risk, and coagulopathy. Despite these challenges, advancements in device technology have led to improved outcomes and reduced bleeding rates, establishing ECMO as a viable strategy in managing acute severe dysfunction [6, 7]. A recent meta-analysis describes the prognosis for adult trauma patients requiring ECMO, concluding that it is beneficial for severely traumatized patients by improving their prognosis and serving as a valuable tool in managing severe trauma-related cardiorespiratory failure, hemorrhagic shock, and cardiac arrest [8]. The use of ECMO to support the recovery of a patient after a pneumonectomy could be a tool for supporting the heart in right ventricular dysfunction and in cases of developed acute respiratory distress syndrome (ARDS). We present a case of penetrating thoracic trauma necessitating TP, supported by ECMO.

## Case presentation

**Fig. 1:**
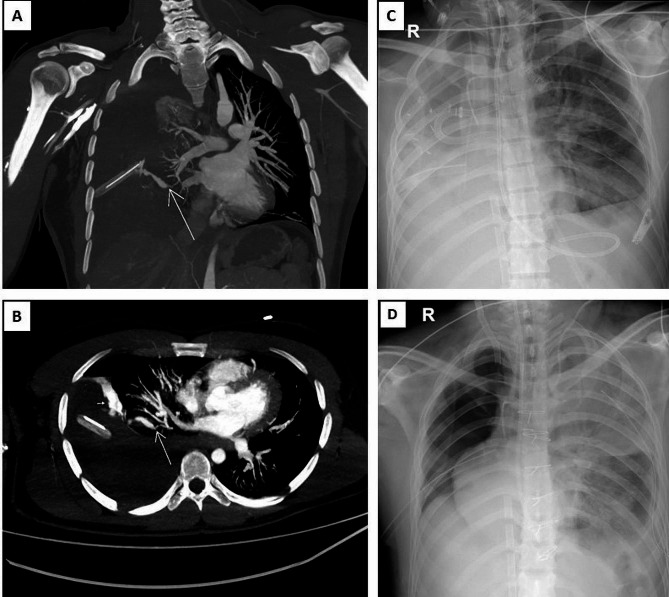
Thoracic Trauma and ECMO support. **A** y **B**. Multiplanar reconstruction with maximum intensity projection. Coronal and axial lung views showing bleeding from a posterior basal subsegmental artery of the lower right lobe (arrow) with extravasation of the contrast medium into the pleural cavity resulting in a massive coagulated hemothorax. **C**. Chest X-Ray shows central ECMO cannulation as a support after lung pneumonectomy. **D**. Chest X-Ray shows peripheral ECMO cannulation as pulmonary support due to respiratory failure

A 34-year-old male, previously healthy, was admitted to the emergency department (ED) following a penetrating thoracic trauma caused by a stab wound. Upon arrival, his temperature was 36.5 °C, pulse 77/min and blood pressure were 60/40 mmHg. Pulse oximetry on room air showed an oxygen saturation of 91%. Examination revealed three stab wounds: the first in the posterior cervical region, the second in the right posterior hemithorax at T4 with a 2 cm scapular line, and the third in the right lumbar region at L1 with a 3 cm wound. Auscultation of the chest showed decreased breath sounds on the right. The eFAST showed right hemothorax. The emergent thoracostomy drained 450 mL of blood. Thoraco-abdominal Angio-CT was performed, ruling out vascular or spinal cord injuries, but finding a massive right hemothorax from an artery of the lower right lobe with active bleeding to the pleural cavity (Fig. 1A, B). At this point the chest tube output reached 1250 ml.

Due to the severity of the situation, the patient was taken to the operative room (OR) for damage control surgery by the trauma surgery team through a midline sternotomy. Intraoperatively, a wound was discovered on the posterior face of the pulmonary hilum that compromised the central portion of the three pulmonary lobes, predominantly affecting the lower lobe, with active bleeding and air leak from the wounds. The pulmonary hilum was clamped to control the bleeding, but due to persistent bleeding and poor oxygenation, a right pulmonary hilum ligation was performed. During the surgery, the patient experienced a cardiac arrest, necessitating direct cardiac compressions in cycles of less than 2 min and a return to spontaneous circulation. Chest tubes, mediastinal packing and delayed sternal closure using the VAC® therapy system was left in place.

The patient was transferred to the Intensive Care Unit (ICU), where his condition deteriorated, presenting with refractory multiple organ dysfunction unresponsive to vasopressor support, respiratory failure characterized by severe hypoxemia and restrictive disorder, and severe hypercapnia. A transesophageal echocardiogram (TEE) was performed, revealing significant right ventricular dilatation and septal flattening, with underfilled left cardiac chambers due to impaired filling of the left heart—indicative of severe right ventricular dysfunction that progressed to cardiogenic shock (Table 1; Hour 0). Consequently, the intensivist team and the ECMO support group decided to initiate peripheral veno-arterial (VA-ECMO) using a femoro-femoral configuration.

**Table 1:** Daily Record of Hemodynamics, Vasopressor Support, Ventilatory Parameters, Drainage, and ECMO Status Over the First Eight Days in ICU

	*Day 0: Hour 6	Day 0: Hour 12	Day 1: Hour 24	Day 1: Hour 36	Day 2	Day 3	Day 4	Day 5	Day 6	Day 7
**Hemodynamic Parameters**										
Heart Rate (bpm)	96	131	132	126	121	71	74	76	91	85
Arterial Pressure (mmHg)	95/80	73/59	100/52	83/63	92/56	60/56	80/64	81/65	101/72	107/82
Hemoglobin (mg/dL)	9,8	12,4	11,4	10	8,6	10,1	8,9	9,5	9,9	10,8
Platelets (per µL)	151,000	80,000	66,000	26,000	31,000	51,000	67,000	59,000	69,000	80,000
pH	7.2	7.21	7.07	7.43	7.41	7.47	7.37	7.34	7.42	7.47
pCO2 (mmHg)	35	42	57	37	42	37	48	48	37	36
pO2 (mmHg)	110	87	109	91	66	137	66	140	130	112
HCO3 (mEq/L)	17.5	16.8	16.5	25	26.3	26.9	27	25	24	25
BE (mEq/L)	-11	-10.6	-13.6	0.3	12	3.2	2.4	0.1	0	1
Lactate (mmol/L)	7.41	6.4	5	3.3	3.7	2.3	0.9	0.6	1	0.9
Creatinine (mg/dL)	1.18	0.97	1.8	2.2	2.3	2.29	2.6	2.5	1.72	1.4
Bilirubin (mg/dL)	-	-	1.15	1.8	3.16	3.25	2.2	2.9	4.4	4.2
**Vasopressor Support**										
Norepinephrine (mcg/kg/min)	0,5	0,343	0,343	0,343	0,343	0,0067	0,026	0,026	-	-
Vasopressin (UI/min)	-	0,047	0,047	0,0633	0,047	0,04	0,04	0,06	-	-
Dobutamine (cc/hr)	-	-	6,4	-	10		-		-	-
Milrinone (cc/hr)	-	-	-	13,5	13,5	13,5	13,5	11,8	16,6	16,6
**Ventilatory Parameters**										
Respiratory Rate	22	22	35	10	10	10	10	10	11	12
Oxygen Saturation	100	94	82	99	92	99	98	99	99	98
Ventilation Mode	VC	VC	PC	PC	APC	PC	PC	PC	APC	PC
FiO2 (%)	0.40	50	100	30	30	30	30	30	30	30
IMV (times/min)	16	22	35	10	10	10	10	10	10	10
PEEP (cm H2O)	10	10	10	10	10	10	10	10	10	8
Tidal Volume (mL)	480	430	260	221	110	110	111	200	471	360
**Drainages**										
Left Thoracic Tube (cc)	-	750	1372	1850	2250	700	400	253	120	420
Right Thoracic Tube (cc)	-	430	805	850	900	250	300	470	420	418
VAC (cc)	-	150	400	500	700	300	350	350	0	500
**ECMO Record**										
Sweep Gas Flow (SGF) L/min	-	-	5,6	6,1	5,5	4	6,96	8	8,25	8
ECMO Flow (L/min)	-	-	4,5	4,0	4,0	3,6	4,8	4,8	3,6	4,0

*Day 0: Refers to the day of the trauma, 6 h post-admission to the ICU following damage control surgery

Three hours after VA-ECMO cannulation, the patient exhibited signs of ischemia in the lower right limb, indicated by asymmetric Near-Infrared Spectroscopy (NIRS) readings. He was promptly taken back to the OR. Initially, switch to central VA-ECMO support using a femoral and ascending aorta configuration. Subsequently, a femoral cutdown was performed, involving the removal of arterial cannula, followed by an arterial embolectomy, which restored perfusion to the right lower limb. Finally, the thoracic surgery team performed a right pneumonectomy (Fig. 1C).

During the ICU stay, veno-venous hemofiltration and dual vasopressor support were necessary, along with multiple blood transfusions. The hemodynamic, ventilatory parameters, vasopressor support, drainages and ECMO record during the first eight days of VA-ECMO cannulation are detailed in Table 1. The characteristics of the timeline, surgical procedures and ECMO cannulation configurations are presented in Table 2.

**Table 2:** Key Events and Interventions of the patient

Day	Hour	Event	Procedure Details
0	0	Damage Control Surgery	
0	6	Admission to ICU after damage control surgery	
1	29	Initiation of peripheral VA-ECMO	Femoro-femoral cannulation (15–25 French)
1	33	Initiation of central VA-ECMO	Aorto-femoral cannulation (17–25 French)
		Femoral Arterial Embolectomy	
		Pneumonectomy	
7		Switch to VV-ECMO	Jugulo-femoral cannulation (18–25 French)
		Sternal Closure	
11		Removal of Right Thoracostomy	
12		Percutaneous Tracheostomy	
61		Removal of VV-ECMO Support	
112		Transfer to Intermediate Care Unit (UCIN)	
124		Transfer to General Ward	
133		Discharge to Home with Home Care Services	

Sternal closure and successful switch from central VA-ECMO to peripheral veno-venous (VV-ECMO) were achieved on the 7th day, due to cardiac function recovery and to prevent hypoxemia and acute lung or respiratory failure (Fig. 1D). On the 11th day, the right thoracostomy was removed, and on the 12th day, he underwent a percutaneous tracheostomy.

The patient experienced several episodes of bronchial obstruction due to thrombus formation, which were treated with bronchoalveolar lavage. He also developed pneumonia caused by multidrug-resistant *Pseudomonas aeruginosa*, necessitating antimicrobial therapy. On the 61st day, the patient was successfully weaned off VV-ECMO but required oxygen support with high-flow nasal cannula for three additional days.

Subsequently, he required oxygen support with venturi mask and was transferred to the intermediate care unit on day 112. Then, he was transferred to a general hospitalization ward for complete recovery, resulting in a total of 133 days of in-hospital care. Table 4 describes the timeline of hospitalizations. On day 133, the patient was discharged with home care arrangements.

## Discussion and conclusions

This case involves a young male with penetrating trauma who was admitted to the ED hemodynamically unstable. A CT scan revealed the source of bleeding in the right hemithorax. He was taken to the OR for a median sternotomy approach for damage control surgery. There was a major lesion from the right pulmonary hiluim profusely bleeding, necessitating clamping and ligation of the right pulmonary hilum, packing of the thorax, and transfer to the ICU for stabilization before completing pneumonectomy. Subsequent complications from the hilum ligation were managed with dynamic hybrid ECMO support, leading to the patient’s recovery and survival from a fatal injury.

The ligation of the hilium and posterior TP carries a high mortality rate. The postoperative course is also associated with a rate of 72% of serious pulmonary, pleural space and cardiovascular complications [1, 7]. The patient exhibits right ventricular failure and pulmonary edema with severe acute hypoxemia. This pulmonary edema is known as post-pneumonectomy pulmonary edema, described very rarely and associated with mortality rates such as 80 to 100% [9]. VA-ECMO supports lung rest while keeping low mechanical ventilator parameters [10]. Once the right heart adapted, VA-ECMO was no longer needed.

In our experience, the decision to defer pneumonectomy is based on data from hospitals in Cali, which show a significantly reduced mortality rate of less than 40% when pneumonectomy is postponed in unstable patients [11]. This delay allows time to correct hypothermia, acidosis, and coagulopathy, which are crucial for patient survival. At that time, our institution did not have ECMO support available; notably, the only patient who died following pneumonectomy suffered from severe pulmonary hypertension, a condition that could potentially have been managed with ECMO. During the damage control surgery, the patient was exsanguinated and very unstable. Immediate ligation and transfer to the ICU were critical for stabilizing the patient over the next 24 h, allowing for a second operation to complete the pneumonectomy [4]. This approach provided time to improve metabolic homeostasis. Recent meta-analyses indicate that ECMO is beneficial for severely traumatized patients by improving their prognosis and serving as a valuable tool in managing severe trauma-related cardiorespiratory failure, hemorrhagic shock, and cardiac arrest [8].

ECMO support is also associated with numerous complications [9], such as thrombosis or embolia that require blood products to correct coagulopathy. In this patient, lower limb ischemia was present, however it was related to femoral/arterial cannula ratio. Stroke is a major concern; however, we achieved a balance between thrombosis and coagulopathy and had no neurological events. In this instance, an infectious complication arises from ventilator-associated pneumonia (VAP) caused by multidrug resistant *Pseudomonas aeruginosa.* A dynamic hybrid ECMO configuration was considered, and the patient was converted to VV-ECMO support until the pneumonia resolved. Dynamic configurations in severe cases have been associated with a lower incidence of mortality in high-risk patients [12].

In conclusion, penetrating thoracic injuries with severe pulmonary vascular lesions sometimes necessitate a pneumonectomy. ECMO support presents as an alternative that can reduce the associated mortality by offering support during the adaptation to pathophysiological cardiopulmonary changes. A multidisciplinary team is essential to minimize complications associated with pneumonectomy, ECMO use, and prolonged hospital stays.

## Data Availability

No datasets were generated or analysed during the current study.

## Abbreviations

TP: Traumatic pneumonectomy
ECMO: Extracorporeal Membrane Oxygenation
CT: Computed Tomography
ICU: Intensive Care Unit
ARDS: Acute Respiratory Distress Syndrome
VA: ECMO-Venoarterial Extracorporeal Membrane Oxygenation
OR: Operating room
CPB: Cardiopulmonary bypass
VV: ECMO-Venovenous extracorporeal membrane oxygenation
VAP: Ventilator-associated pneumonia
